# Age Affects How Task Difficulty and Complexity Modulate Perceptual Decision-Making

**DOI:** 10.3389/fnagi.2019.00028

**Published:** 2019-03-01

**Authors:** Claudine Habak, Mohamed L. Seghier, Julie Brûlé, Mohamed A. Fahim, Oury Monchi

**Affiliations:** ^1^Cognitive Neuroimaging Unit, Emirates College for Advanced Education, Abu Dhabi, United Arab Emirates; ^2^Centre de Recherche, Institut Universitaire de Gériatrie de Montréal, Université de Montréal, Montreal, QC, Canada; ^3^School of Optometry, Université de Montréal, Montreal, QC, Canada; ^4^Department of Clinical Neurosciences, and Radiology, University of Calgary, Calgary, AB, Canada; ^5^Hotchkiss Brain Institute, Cumming School of Medicine, University of Calgary, Calgary, AB, Canada

**Keywords:** aging, decision-making, perceptual discrimination, task difficulty, task complexity, fMRI signal modulation, functional MRI, psychophysics

## Abstract

Decisions differ in difficulty and rely on perceptual information that varies in richness (complexity); aging affects cognitive function including decision-making, and yet, the interaction between difficulty and perceptual complexity have rarely been addressed in aging. Using a parametric fMRI modulation analysis and psychophysics, we address how task difficulty affects decision-making when controlling for the complexity of the perceptual context in which decisions are made. Perceptual complexity was varied in a factorial design while participants made perceptual judgments on the spatial frequency of two patches that either shared the same orientation (simple condition) or were orthogonal in orientation (complex condition). Psychophysical thresholds were measured for each participant in each condition and served to set individualized levels of difficulty during scanning. Findings indicate that discriminability interacts with complexity, to influence decisional difficulty. Modulation as a function of difficulty is maintained with age, as indicated by coupling between increased activation in fronto-parietal regions and suppression in the lateral hubs, however, age has a specific effect in the ventral anterior cingulate cortex (ACC), driven by performance at near-threshold (difficult) levels for the simpler stimulus combination condition, but not the more complex one. Taken together, our findings suggest that the context of difficulty, or what is perceived as important, changes with age, and that decisions that would seem neutral to younger participants, may carry more emphasis with age.

## Introduction

As we age, changes arise in visual perception (e.g., Sekuler and Sekuler, 2000; Owsley, 2011) and in cognitive function across domains (e.g., Buckner, 2004; Craik and Bialystok, 2006). We make countless decisions of varying difficulty that rely on perceptual information, and yet, there has been little focus on the interaction between lower-level visual content and higher-level decision-making in aging. This fMRI study investigates how general task demand and complexity of visual content modulate perceptual decision making in aging.

Tasks of executive control and decision-making involve prefrontal cortex (Rajah et al., 2008) along with fronto-striatal interactions when task demands increase (Rogers et al., 2000; Monchi et al., 2001, 2006). With age, cognitive tasks elicit broader activation of anterior executive regions, including pre-frontal cortex (Reuter-Lorenz and Lustig, 2005; Mattay et al., 2006; Cappell et al., 2010), and the timing of this recruitment can shift to maximize information usage (Velanova et al., 2007; Martins et al., 2012). In addition, general patterns of network change, display higher activation of visual networks and lesser activation of control and default-mode networks with age (Li et al., 2015), along with reduced connectivity in default and control networks (Grady et al., 2016). Task demand, or cognitive difficulty, modulates brain activity whose relative activations and deactivations link to behavioral performance. In aging, when difficulty increases, modulation decreases in a fronto-parietal network and in ventromedial prefrontal cortex; furthermore, preservation of the relative pattern of activation and deactivation (balance between positive and negative modulation) is associated with better performance in aging (Kennedy et al., 2015; Rieck et al., 2017). Tasks of executive function such as decision-making, generally rely on one type of difficulty, but task demands can arise from various sources and can be manipulated by visual perceptual content.

Visual function exhibits widespread effects of age, reflecting changes in neural processing at various stages of the visual stream (for reviews, see Spear, 1993; Sekuler and Sekuler, 2000; Owsley, 2011). It is widely accepted that the cortical representation of visual features follows a hierarchy, where information from lower-level mechanisms is integrated at ensuing stages to yield progressively more complex representations. Beginning with modules for simple features such as line orientation and spatial frequency in early visual cortex – V1 (Hubel and Wiesel, 1968; De Valois and De Valois, 1990), progressing through combinations across orientations in V2, to curvature and shape in V4 (Gallant et al., 1993, 1996; Kobatake and Tanaka, 1994; Pasupathy and Connor, 1999, 2001), and culminating with face representation in temporal cortex (Kobatake and Tanaka, 1994; Brincat and Connor, 2004), which is distributed across multiple modules (Grill-Spector et al., 1999; McIntosh et al., 1999; Haxby et al., 2000; Vuilleumier et al., 2002; Andrews and Ewbank, 2004). With age, an interesting pattern arises: performance is stable when stimulus processing relies on operations within a visual module but deteriorates when a greater number of operations across modules is involved, and this, for various representations including the perception of motion (Habak and Faubert, 2000), texture (Habak and Faubert, 2000; Habak et al., 2009), shape (Habak et al., 2009), and faces (Habak et al., 2008). This age-related performance decline seems linked to the complexity of the visual operations and not to general task demands alone, as it appears even when tasks are alike (Habak and Faubert, 2000; Habak et al., 2009). These performance differences linked to communication within and among modules in a network, are analogous to recent work on age-related changes in functional network interactions, which with age, display reduced functional connectivity within networks, such as within the default-mode network, or within the fronto-parietal control network (Grady et al., 2016). This provides a framework by which the interaction of difficulty arising from perceptual content and from task demand can be addressed in aging.

The purpose of the present work is to address the effect of aging on difficulty arising from general task demands and difficulty arising from visual complexity in a factorial design, by asking participants to make perceptual judgments on the spatial frequency of two patches. General task demand is manipulated by varying the discriminability of two patterns along the spatial frequency dimension (levels of task difficulty), and visual complexity is controlled by changing the relative orientation of the pattern-pair (levels of task complexity). According to models of the neural substrates of vision, patterns that share the same orientation would involve mainly V1, whereas comparison across two different orientations would also involve V2 and V4 (Lin and Wilson, 1996; Fang et al., 2005; Allen et al., 2007), thus involving more modules, and should show a stronger effect of age. Analogously, in younger participants, spatial-frequency discrimination between two patterns in visual memory elicits higher activation in V1 and V2 for different orientations than for same orientations (Baumann et al., 2008). We chose these patterns because their processing mechanisms are well documented in visual perception, discriminability can be equated for each individual observer, and semantic content is minimal. This allows us to look at the effect of perceptual complexity on cognitive decision function, i.e., the effect of changes in posterior brain representation on anterior function, when difficulty is controlled. We expect the interaction between general task demand and perceptual complexity to differ with age, with significant effects in anterior brain regions.

Because (1) we were interested in relative differences in the interaction between task demand and complexity, and (2) the experiment manipulated a number of discriminability/difficulty levels, a parametric modulation fMRI analysis was better suited to our mixed design. This allows activity changes to be assessed beyond the standard subtraction logic of task-based fMRI analysis. The behavioral task we used measures discrimination performance (correct responses) along with response time for each of the four discriminability levels and the two conditions of complexity; generally, longer response times are associated with more difficult decisions.

## Materials and Methods

This study was approved by the Research Ethics Committee of the Regroupement Neuroimagerie Québec. All participants provided written informed consent to participate in this study in accordance with the Declaration of Helsinki.

### Participants

Twenty right-handed adults participated in this study, with twelve younger (6 female, 6 male, age 28.4 ± 3 years, range: 24–32) and eight older participants (4 female, 4 male, age 68.4 ± 7 years, range: 62–82). They had normal or corrected-to-normal vision, and no history of neurological disorders. All participants were screened for depression using the Beck Depression Inventory (score ≤ 16). Older participants were screened for Mild Cognitive Impairment using the Montreal Cognitive Assessment (Nasreddine et al., 2005); criterion of inclusion: score ≥ 26) and underwent a complete eye examination by an optometrist to ensure good acuity (better than 20/25, corrected), along with good ocular health and function. Participants wore appropriate correction during the psychophysical and scanning sessions.

### Stimuli

**FIGURE 1:**
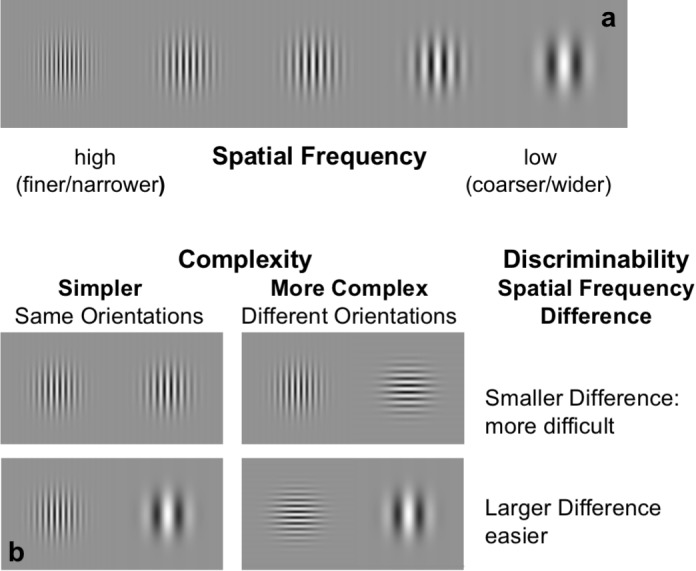
Stimuli and design. **(a)** Gabor patch stimuli (sine-wave carrier in a Gaussian window) with examples of different sine-wave frequencies. **(b)** Example of discriminability levels: participants were asked to specify which pattern of the pair had the highest spatial frequency (finer/narrower lines) for various levels of difference. Top pairs: illustrate low-discriminability (less difference, more difficult), bottom pairs: illustrate high discriminability (more difference, easier). Left panel pairs: same orientation condition (lower complexity), right panel pairs: different orientation conditions (higher complexity).

Stimuli were generated using custom Matlab software, incorporating routines from the Psychophysics and Video Toolbox (Brainard, 1997; Pelli, 1997). They consisted of two Gabor patches presented side-by-side that remained on-screen until observers responded (Figure 1). Gabor patches consist of a circular Gaussian window (envelope) multiplied with a sine-wave carrier. Gaussian windows had a standard deviation of 0.28 and the sine-wave carrier of one patch, a period of 0.75 cpd. This frequency was randomly jittered by ±10% from trial to trial and served as the reference. The carrier spatial frequency of the other patch was randomly increased or decreased from the ±10%-reference by one of 6 Weber Fraction levels (0.0125, 0.025, 0.05, 0.1, 0.2, 0.4). The side (left or right-hand side patch) on which the reference frequency appeared, was randomized from trial to trial. In addition, carrier phase was randomly jittered from trial to trial independently for each patch, to avoid participant discrimination based on a single point of the image. Participants were instructed to fixate the center of the screen, but a fixation point was not provided, because this could serve as a reference against which spatial-frequencies could be compared. Stimulus luminance contrast was 30%.

### Out-of-Scanner Behavioral Experiment

This experiment aimed to measure the spatial frequency discrimination thresholds (i.e., performance) in each participant. Two conditions were tested: a same-orientation condition, in which carrier orientation in each of the two patches was identical (fixed at vertical) and a different-oriented condition, in which carriers were oriented orthogonally at 90° to each other (one vertical and one horizontal). In the different-oriented (i.e., orthogonal) condition, carrier orientation (vertical or horizontal) was varied randomly between the two patches from trial to trial. Experiments were conducted using the method of constant stimuli and a two-alternative forced-choice (2-AFC) procedure, in which participants indicated which of the two patches contained the higher spatial frequency (finer lines); no feedback was provided. Each level was shown 20 times in pseudo-random order, for a total of 120 trials per block. Orientation conditions (same vs. different) were tested in separate blocks, and each was tested a minimum of 2 times. In the test blocks used to measure discrimination thresholds, 5–6 levels of spatial frequency difference were used. Orientation conditions were not interleaved but blocked, to avoid monitoring of multiple orientation channels (Graham, 1989) during the same orientation condition: if participants monitored more than one orientation channel, then the same- and different-orientation conditions would be effectively similar. For each trial in a block, stimuli appeared and remained on-screen until participants responded, and trials were separated by a 400 ms interval (inter-trial interval, ITI); participants were informed if the block consisted of same-orientation or orthogonal-orientation conditions. A session totaled a minimum of 600 trials. At least one practice block of 30 trials per condition was given prior to testing.

Measures from each block were fit with a sigmoidal Quick (1974) or Weibull (1951) function using maximum likelihood estimation, and discrimination threshold was defined at 75% correct responses. Response time, from stimulus onset to response key-press, was also recorded.

Response times and not speeded reaction times, were measured for each trial, where participants are not instructed to respond as rapidly as possible. For both in- and out-of-scanner sessions, participants were instructed to make discriminations: they were informed that we sought points of individual discriminability, so some discriminations would appear easier and some more difficult, and to simply respond when they could; no mention of speeded responses was made.

All stimuli were presented on a contrast-linearized CRT with a refresh rate of 75 Hz. The monitor was viewed binocularly from a distance of 90 cm (resolution 1024 × 768), so that one pixel subtended the same visual angle as a pixel viewed through the mirror on the projected screen in the scanner (1.1 arcmn). In the scanner room, projector resolution was 1024 × 768; the image was rear-projected onto a 57 × 45.5 cm screen, subtending 19.1 × 14.3 degrees of visual angle from the viewing distance of 170 cm, so that one pixel subtended 1.1 arcmn.

### fMRI Experiment

Conditions were identical to the behavioral session, but only four levels of spatial frequency difference were used. Based on the behavioral psychophysics session, levels of spatial frequency difference between the two patches (Weber Fractions) were set to yield correct responses of approximately 50, 75, 85, and 100% for each individual participant. Levels of spatial frequency difference (i.e., levels of discriminability) defined our four levels of task difficulty, from very difficult at 50% correct responses or chance, to very easy at 100% correct responses. This was carried out separately for each condition (same vs. different orientations), as discrimination thresholds were higher across different (90°) orientations. Again, no feedback regarding responses (correct or incorrect) was provided. As for the out-of-scanner behavioral experiment, response times and not speeded reaction times, were measured for each trial.

In a mixed event-related design, one run consisted of four blocks, where two blocks per orientation condition were shown. Each block contained 40 trials (i.e., 10 trials per difficulty level) of a given orientation condition (same or different) for a total of 160 trials per run. Each trial/event began 2 s after response to the previous image, with the constraint that a minimum of 3000–3300 ms (randomized) elapsed from stimulus onset, and there was an additional 400 ms inter-trial-interval before appearance of the next trial. At the beginning of each block, after 1.5 s of mean gray, a cue appeared for 3 s to indicate whether the block would consist of same orientations (a black //) or different orientations (a black X), for a total of 6 s, and the cue was followed by 1.5 s of mean gray before trials began (Figure 1b). Participants were instructed to fixate the center of the screen. Block order in each run consisted of ABAB or BABA and was counterbalanced across runs and across participants. Response choice, along with response time (RT) defined as the time between stimulus onset and response button press, were recorded while participants were in the scanner.

### MRI Acquisition

Experiments were performed on a 3T Siemens TIM system (Siemens Medical Systems, Erlangen, Germany) at the Institut Universitaire de Gériatrie de Montréal. Functional imaging consisted of an EPI GRE sequence (TR/TE/Flip = 2500 ms/30 ms/90°, FOV = 192 mm, matrix = 64 × 64, 41 axial slices, 3.5 mm thick with no gap, isotropic voxels of 3.5 × 3.5 × 3.5 mm^3^). To minimize head motion at the time of data acquisition, prospective motion correction (“pace”) was used by measuring participant motion in real time and dynamically updating the imaging sequence (Thesen et al., 2000). Functional scanning was always preceded by dummy scans to ensure tissue steady-state magnetization. Functional images were acquired over four runs in a single session. High-resolution anatomical T1-weighted images were acquired using a three-dimensional gradient-echo sequence (spatial resolution of 1 mm^3^) at the beginning of the session, prior to the functional scans.

### fMRI Data Analysis

Data processing and statistical analyses were performed with the Statistical Parametric Mapping software package, using the latest version SPM12 (Wellcome Trust Centre for Neuroimaging^1^, London, United Kingdom). Preprocessing was carried out with standard SPM analysis procedures: all functional volumes were spatially realigned, un-warped, slice-timing corrected, normalized to MNI space using the new unified normalization-segmentation procedure of SPM12, and smoothed with an isotropic 8-mm FWHM Gaussian kernel, with resulting voxel size of 2 × 2 × 2 mm^3^. The pre-processed functional volumes of each participant were then submitted to a fixed-effects analysis (first-level analysis), using the general linear model at each voxel. Each stimulus/trial onset was modeled as an event in condition-specific “stick-functions” with a duration equal to the self-paced responses in each participant (Grinband et al., 2008). For each stimulus orientation (same or different), task difficulty was included as a single linear parametric modulator that was orthogonal to the main trial regressor, so that we could look at effects beyond the categorical subtractive logic of fMRI. This parametric modulation allowed us to separate the main effect of perceptual decision making (i.e., any kind of visual discrimination) from the impact of difficulty when varying stimulus spatial frequency differences. Resulting stimulus functions were convolved with a canonical hemodynamic response function, which provided the regressors for the linear model. For each participant, a contrast image was generated to summarize the effect of response modulation by task difficulty for each of the two stimulus orientation conditions.

For the group analysis, contrast images of the parametric modulation by difficulty at the two stimulus orientation conditions (same and different orientations), for each of the 20 participants’ first-level analyses, were entered into a second-level factorial analysis in SPM (i.e., random-effects analysis, Factor 1 = group, Factor 2 = stimulus orientation). From this second level group analysis, statistical parametric maps of the SPM{t} statistic were generated at each voxel SPM{t} for the differences in parametric modulation and the interaction between age and stimulus orientation. Statistical comparisons are reported at a threshold of *p* < 0.05 FWE-corrected for height or size across the whole brain.

## Results

### Psychophysics

For younger participants, psychophysical baseline tasks yielded mean spatial-frequency difference discrimination thresholds of 0.054 ± 0.004 (SEM) for the same-orientation (lower complexity) condition and of 0.077 ± 0.007 for different orientations (higher complexity), with overall response times (collapsed across all levels) of 1668.7 ms ± 171.3 and 1516.7 ms ± 128.6, for each condition, respectively. For older participants, psychophysical baseline tasks yielded mean spatial-frequency difference discrimination thresholds of 0.069 ± 0.014 for the same-orientation condition and of 0.14 ± 0.024 for different orientations, with overall response times (collapsed across all levels) of 2046.7 ms ± 211.2 and 2694.0 ms ± 297.3, for each condition, respectively. For discrimination thresholds, a two-way repeated measures ANOVA indicated a main effect of orientation condition (*F*_1,18_ = 19.2, *p* < 0.001) and an interaction between age group and orientation condition (*F*_1,18_ = 4.65, *p* = 0.045), with no main effect of age. The interaction was driven by higher thresholds for the different-orientation condition in older participants. Stimuli used in the scanner were equated for difficulty for each orientation condition and for each individual participant, based on their individual thresholds during psychophysical testing.

### Behavioral Results in Scanner

Analysis of variance on response times confirm the expected main effect of difficulty arising from stimulus similarity (*F*_1,18_ = 163.6, *p* < 0.001; Table 1), with slower responses in older participants (*F*_1,18_ = 64.6, *p* = 0.004), but no effect of stimulus complexity (orientation condition *F*_1,18_ = 0.12, *p* > 0.1). The only significant interaction was between age and similarity level (*F*_1,18_ = 11.5, *p* = 0.04), driven by slower response times in older participants when perceptual decisions were made at discrimination threshold levels (75% correct responses; Table 1). By design, in scanner responses should have yielded correct responses of approximately 50, 75, 85, and 100% at each of the four levels of difficulty for each of the complexity conditions. Accordingly, Table 1 shows that in-scanner responses yielded matching values, but for the different orientation condition in both groups of participants, performance at the expected 85% level (level 3) exceeded that setting with >90% correct responses; the same occurred for older participants at that level in the same-orientation condition. Similar to response-time analyses, analysis of variance on correct responses (accuracy) confirm the main effect of discriminability arising from stimulus similarity (*F*_1,18_ = 451.2, *p* < 0.0001), with slightly better responses in older participants (*F*_1,18_ = 11.23, *p* = 0.004), and a borderline effect of orientation condition (*F*_1,18_ = 4.53, *p* = 0.047).

**Table 1:** In-scanner mean performance in percent correct responses and response times in milliseconds (±1SEM) for both older and younger groups, for each orientation combination (task complexity) and each discriminability level (task demands).

Orientations Task Complexity	Discriminability levels Task Demand		Performance % correct responses ± 1SEM		Response Time ms ± 1SEM	
			Younger	Older	Younger	Older
**Same**	**Most similar**	**Level 1**	52.2 ± 1.27	55.1 ± 1.69	1849 ± 113	2182 ± 187
Simpler	Most difficult	50%				
		**Level 2**	71.8 ± 2.58	71.2 ± 3.81	1672 ± 85	2339 ± 277
		75%				
		**Level 3**	88.3 ± 2.06	95.6 ± 1.88	1514 ± 94	1670 ± 178
		85%				
	**Least similar**	**Level 4**	99.5 ± 0.26	99.7 ± 0.22	1015 ± 55	1088 ± 64
	Easiest	100%				
**Different**	**Most similar**	**Level 1**	53.8 ± 2.11	56.0 ± 2.21	1872 ± 97	2358 ± 259
More Complex	Most difficult	50%				
		**Level 2**	72.8 ± 2.36	79.2 ± 1.84	1746 ± 87	2259 ± 245
		75%				
		**Level 3**	91.0 ± 2.13	98.6 ± 0.39	1405 ± 69	1425 ± 134
		85%				
	**Least similar**	**Level 4**	99.2 ± 0.63	99.5 ± 0.24	1028 ± 30	1135 ± 52
	Easiest	100%				

*Discriminability levels are indicated by a scale of 1 to 4 with corresponding performance settings in % correct responses.*

### Imaging Results

**FIGURE 2:**
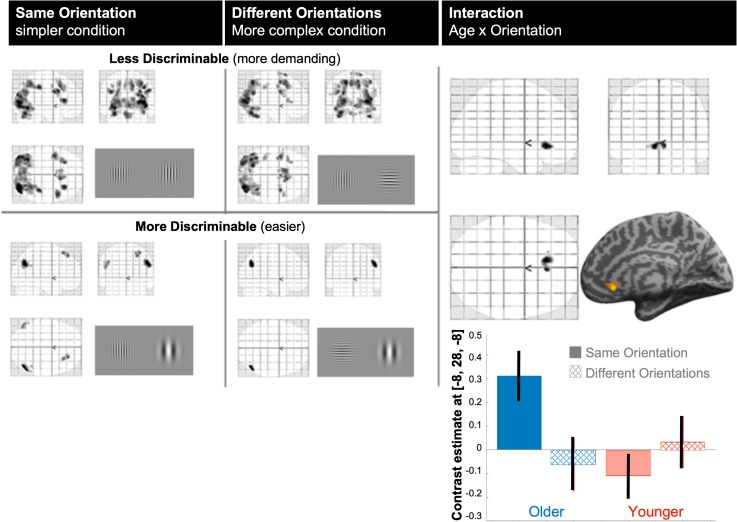
BOLD modulation. The left panel shows main mean effects of parametric modulation by discriminability (task demand) for each stimulus orientation condition (complexity): the simpler, same-orientation condition, and the more complex, different orientation condition. Top row “less discriminable” indicates regions where BOLD modulation increased with task demand (illustrated by stimuli difficult to discriminate); bottom row “more discriminable” indicates regions where BOLD modulation decreased with task difficulty (illustrated by stimuli easy to discriminate). All significant effects were projected on the standard glass brain of SPM in MNI space. All maps are shown at *p* < 0.05 FWE-corrected for size or height; Right panel: the only cluster that was significant in the age by orientation interaction was located in the ventral anterior cingulate cortex [MNI coordinates = –8, +24, –8]. Below, contrast estimates (size of modulation) with 90% confidence intervals in this ACC cluster, shows that the interaction arose from the same-orientation condition.

Figure 2 illustrates the modulation of brain activity by demand (discriminability difficulty) for the two complexity conditions (same or different stimulus orientations). At corrected *p*-FWE < 0.05, brain activity modulation increased significantly with task demand (positive parametric modulation over all participants) for both orientation conditions in different bilateral occipital, parietal and frontal regions, whereas brain responses in bilateral inferior parietal lobule decreased with task difficulty. The latter was due to stronger suppression of those posterior lateral hubs of the default mode network when task difficulty increased for both orientation conditions. The main effect of age over the two orientation conditions and the main effect of orientation condition over the two groups were not significant (at *p* < 0.001 uncorrected). However, there was a significant interaction (*p* < 0.05 FWE-corrected, Figure 2) between age and orientation (complexity) condition in the subgenual anterior cingulate cortex (ACC, MNI-coordinates[–8, +28, –8], *Z*-score = 4.7, *k* = 162 voxels), indicating that differences in task difficulty modulation between the two stimulus orientation conditions (same or different) differed between our older and younger participants. Interestingly, this interaction was driven by a significant difference between older versus younger participants for the same orientation, or less complex condition (*Z*-score = 4.5), as illustrated by the effect-size bar graph in Figure 2 (bottom-right).

## Discussion

This work shows that discriminability interacts with complexity, to influence decisional difficulty, and that age has a specific effect at near-threshold levels for simpler stimulus combinations in the ventral ACC. Performance was individually matched for each observer and complexity condition, so effects could not arise from performance biases. Discriminability served as a modulator, allowing us to look at relative changes in brain activation as a function of decisional difficulty (complexity/discriminability). Interestingly, the age-related differences in modulation between orientation conditions arose not in posterior perceptual regions, but rather in an anterior region (ACC), pointing to the interaction between perceptual information and decisional processing.

The finding that age affects brain activity modulation as a function of discriminability in the ventral ACC is particularly interesting. This effect could be driven by at-threshold, or difficult, discrimination in the same orientation condition (simpler orientation condition), as shown in the behavioral analysis. Interestingly, in younger participants, activity of the ACC increases when discriminability and confidence increase for a perceptual decision (Rolls et al., 2010), and in aging, reduced function through metabolic activity occurs in the anterior attentional network involving the ACC, including the subgenual ACC (Pardo et al., 2007). These studies mainly reported changes in mean activity from baseline, but our data address modulation of activity by difficulty. A better fit to our findings comes from the context of possible age-related changes in the valence of the interaction between stimulus configuration and decisional difficultly. Generally, the dorsal ACC is involved in the cognitive aspects of decision-making, likely related to evaluating the outcome of choices (Botvinick et al., 2004; Walton et al., 2007), whereas the ventral ACC is involved in affect (Phan et al., 2002; Vogt, 2005) or reward-based aspects of decision-making, integrating emotion and cognitive control (Cromheeke and Mueller, 2014; Palomero-Gallagher et al., 2015). Furthermore, in aging, the resting-state functional connectivity of the ACC undergoes reorganization between rostral and dorsal ACC, and between the ACC and other regions to maintain response salience (Cao et al., 2014), emotional stability correlates with increased activation in rostral ACC (Brassen et al., 2011), consistent with maintaining positive affect and cognitive function in successful aging (Brassen et al., 2011; Mather, 2012). We propose that the activation of the ventral (subgenual) ACC here, likely arises from valence attributed to the discrimination by older observers. Discriminability was equated for each individual participant, and accordingly, response time decreased with decreasing difficulty across participants and conditions. However, for same-orientation at-threshold discriminations, older participants displayed longer decision times than below-threshold discriminations (cf. Table 1), which display chance performance and usually exhibit the longest decision times (e.g., as is the case for the different-orientation condition). However, longer response-time alone is not the driving factor, because equally long response times were found for the different-orientation condition at chance levels, and yet there was no ACC-effect for the different-orientation condition. The longer at-threshold decision time for this same-orientation condition, suggests that older participants ascribed more weight to this decision.

The source of this valence most likely arises from a combination of uncertainty and discriminability-difficulty when a decision is plausible. Even though all the stimuli used here are highly visible, near-threshold spatial-frequency differences are discriminable but appear almost identical in the same-orientation condition, whereas the pair’s general structure is distinct (orthogonal orientations) in the different-orientation condition. Viewing two quasi-identical patterns (same orientations) may lead to higher uncertainty than two patterns that differ in some way (different orientations), even if the dimension is task-irrelevant and discriminability is held constant across conditions. Discriminability-difficulty alone would have yielded similar patterns across orientation-conditions, and uncertainty alone would have yielded similar patterns for at-threshold and below-threshold decisions; neither is the case here. When uncertainty and discriminability-difficulty are taken together, adjusting behavioral response and its inherent brain activity for at-threshold discrimination, could be useful to performance. For example, older participants generally sustain greater task engagement (Forstmann et al., 2011), as supported by decreased variability in electrophysiological activity related to attention across trials during perceptual decision-making (McGovern et al., 2018). In tasks using feedback, motivation can shift with age: older participants try to avoid negative outcomes more than younger participants (Frank and Kong, 2008), and they show higher motivation and goal commitment (West et al., 2005). Interestingly, our findings indicate that a motivational shift may have occurred, even though no feedback was given regarding correct or incorrect responses, suggesting that what is considered difficult or important varies with age.

This age-attributed valence could arise from general age-related performance or criterion biases in decision-making: many older participants express wanting to “do well” in experiments, and responses can tend toward the conservative with age (e.g., Ratcliff et al., 2006; Cassidy and Gutchess, 2015). However, the two-alternative forced-choice procedure used here is criterion-independent (Green and Swets, 1974) and therefore not subject to age-associated criterion biases. In addition, other at- or near-threshold decisions would have shown an effect.

Another plausibility is noise in the aging system, which could lead to exacerbated discrimination difficulty or uncertainty at- or near-threshold: for example, reduced signal-to-noise ratio, and reduced tuning for spatial frequency, orientation, and motion direction have been reported in macaque (Schmolesky et al., 2000; Leventhal et al., 2003; Zhang et al., 2008). If a similar process occurred in aging humans, the increased noise in stimulus representation could make discriminations more demanding, and therefore, add weight to higher cognitive processing. Increased noise and reduced tuning in the aging visual system could lead to the same-orientation condition appearing less similar in orientation, and the different-orientation condition appearing less different. In this scenario, the same-orientation condition could lead to slightly more demands for older participants than for younger ones, while the different-orientation condition could be slightly less demanding with age. This pattern is consistent with our findings, and age-related noise and tuning changes in perceptual representation are likely contributing factors. While this is a plausible mechanism, future work is warranted in this area, because in humans, motion direction discrimination is affected by age (Bennett et al., 2007), but orientation (Delahunt et al., 2008; Govenlock et al., 2009) and spatial-frequency selectivity (Govenlock et al., 2010) are maintained with age. Finally if noise and tuning were sole factors, effects of age would be visible regardless of orientation condition. Globally, age-related increased-noise and reduced-tuning in perceptual systems could lead to differences in representational specificity with age, which would interact with higher function to address task demands. The nature of this interaction could involve the ACC and does not rule out valence.

Wider age-related activation in brain activity has been attributed to various accounts, including compensatory activity and dedifferentiation. Compensation proposes the recruitment of non-task-typical brain networks to support performance (Reuter-Lorenz and Cappell, 2008) while, dedifferentiation reflects a loss of specialization (Baltes and Lindenberger, 1997; Park et al., 2001), illustrated by less specific brain activation (Grady et al., 1994; Cabeza et al., 2004; Park et al., 2004; Cappell et al., 2010; Dennis and Cabeza, 2011).

Our age-dependent effect in the subgenual ventral ACC, could have resulted from compensatory activity, but it is unlikely the sole driving factor, as other discriminations would have elicited this pattern. For certain types of demanding tasks, patterns of brain activity display an age-related shift in terms of timing: for example, during decision planning, patterns of brain activity are maintained with age, but younger participants begin planning their change while receiving feedback, whereas older participants begin planning only after feedback is complete (Martins et al., 2012). This is comparable to earlier work on memory, which indicated that older participants rely on later phases of retrieval involving effort and monitoring and less on the earlier phases of the retrieval process (Velanova et al., 2007). These findings suggest that task load is distributed differently with age to maintain performance. Analogously, our age-related modulation in the ACC and seeming change in task-set importance could reflect distribution of task load. A possible mechanism involves perceptual updating, where insula and ACC activation appear when participants proactively update representations regardless of uncertainty (Stöttinger et al., 2018). In this scenario, older participants could be continuously updating their initial stimulus representations when there is a likelihood or chance for improving performance, as per our behavioral analysis.

Overall, younger and older participants displayed similar modulation of brain activity for discriminability, with difficulty-related increases in occipital, parietal, and frontal regions, and a decrease in bilateral inferior parietal lobule. These parietal areas are involved in the default-mode network (Raichle et al., 2001; Greicius et al., 2003), and the coupling between increased activation in fronto-parietal regions and suppression in the lateral hubs suggests that modulation as a function of difficulty is maintained with age, in accordance with findings of this coupling with age (Prakash et al., 2012). For example, for challenging cognitive tasks the coupling between fronto-parietal activity and default mode suppression generally weakens with age (Turner and Spreng, 2015), but older individuals with greater default mode suppression (Park et al., 2010) or coupling, display better performance (Kennedy et al., 2015; Rieck et al., 2017). This is consistent with both our imaging and psychophysical results. Modulation increased with difficulty across all participants, and behaviorally, there was no difference in performance between younger and older participants for the same orientation condition, along with a slight age-related decline for the more complex different-orientation condition. We used these thresholds to equate difficulty individually for each participant in the imaging tasks and looked at relative patterns of brain activity (modulation) as a function of difficulty, as opposed to changes from baseline.

In terms of limitations, the sample size here is small (i.e., 20 participants in total), but the effect in the ACC is significant (please see results): it is visible on a whole-brain analysis with a conservative corrected *p*-FWE of <0.05 and with parametric modulation as opposed to activation. Any other age-related effects not visible here, would likely have a small effect size or poor consistency across participants (for a discussion, please see Friston, 2012). In terms of region of interest (ROI) predictions, our purpose was to address the interactions between perceptual complexity and general task demands in aging, and to test their significance over the whole brain. We were interested in relative differences in brain responses that would explain observed differences in behavior, and our hypothesis targeted function rather than specific ROIs, as there was no basis to expect an effect in predefined ROIs. In addition, we do not know of similar work in aging, combining complexity and demands in perceptual decision-making, so there were no *a priori* coordinates on which to found an ROI-based hypothesis. Despite the limitations, our findings are novel, interesting, and warrant future work: for example, refining our approach by adding more levels of information complexity, incorporating a larger number of participants to better probe the age-dependent effect in the ACC (and perhaps other brain regions), and extending our paradigm to another perceptual modality.

In summary, this work establishes that brain modulation as a function of difficulty is maintained with age, but that the context of difficulty, or what is perceived as important, changes with age, and involves the subgenual ACC. This could arise from decreased specificity in perceptual representation. The key point here, is that decisions that would seem neutral to younger participants, may carry more emphasis with age, and has implications for various areas. Future investigations could address how interactions between the subgenual ACC and other regions change with age, examine the key connectivity metrics that can explain or predict behavioral modulations related to difficulty vs. complexity, and to look into the link between affect and performance related to task difficulty and decision-making in aging.

## Author Contributions

CH conceptualized the study. CH and OM designed the study. CH and JB acquired the data. CH, MS, MF, and OM analyzed and interpreted the data. CH and MS drafted the work. All authors critically reviewed and approved the final version of the manuscript.

## Conflict of Interest Statement

The authors declare that the research was conducted in the absence of any commercial or financial relationships that could be construed as a potential conflict of interest.

## Footnotes

1 http://www.fil.ion.ucl.ac.uk/spm/
